# (2-Meth­oxy-1,3-phenyl­ene)diboronic acid

**DOI:** 10.1107/S160053680800010X

**Published:** 2008-01-09

**Authors:** Marek Dąbrowski, Sergiusz Luliński, Janusz Serwatowski

**Affiliations:** a Warsaw University of Technology, Faculty of Chemistry, Noakowskiego 3, 00-664 Warsaw, Poland

## Abstract

The mol­ecular structure of the title compound, 2-CH_3_O—C_6_H_3_-1,3-[B(OH)_2_]_2_ or C_7_H_10_B_2_O_5_, features two intra­molecular O—H⋯O hydrogen bonds of different strengths. One of the boronic acid groups is almost coplanar with the aromatic ring, whereas the second is significantly twisted. Mol­ecules are linked by inter­molecular O—H⋯O hydrogen bonds, generating infinite chains cross-linked to form a two-dimensional sheet structure aligned parallel to the (01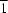) plane.

## Related literature

For structures of other di- and polyboronic acids, see: Fournier *et al.* (2003 ▶); Maly *et al.* (2006 ▶); Pilkington *et al.* (1995 ▶); Rodríguez-Cuamatzi, Vargas-Díaz, Maris, Wuest & Höpfl (2004 ▶); Rodríguez-Cuamatzi, Vargas-Díaz & Höpfl (2004 ▶). For the structural characterization of related *ortho*-alk­oxy aryl­boronic acids, see: Dabrowski *et al.* (2006 ▶); Serwatowski *et al.* (2006 ▶); Yang *et al.* (2005 ▶). For related literature, see: Rettig & Trotter (1977 ▶); Dorman (1966 ▶).
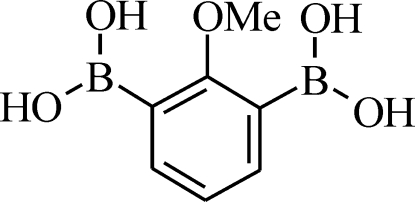


## Experimental

### 

#### Crystal data

C_7_H_10_B_2_O_5_
*M* *_r_* = 195.77
Triclinic, 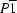

*a* = 5.0261 (6) Å
*b* = 7.6475 (12) Å
*c* = 12.4535 (19) Å
α = 79.010 (13)°
β = 81.898 (12)°
γ = 77.246 (12)°
*V* = 455.85 (11) Å^3^
*Z* = 2
Mo *K*α radiation
μ = 0.12 mm^−1^
*T* = 100 (2) K
0.75 × 0.28 × 0.16 mm

#### Data collection

Kuma KM4 CCD diffractometer
Absorption correction: multi-scan (*CrysAlis RED*; Oxford Diffraction 2005 ▶) *T*_min_ = 0.91, *T*_max_ = 0.98
8626 measured reflections
2191 independent reflections
1884 reflections with *I* > 2σ(*I*)
*R*_int_ = 0.012

#### Refinement

*R*[*F* ^2^ > 2σ(*F* ^2^)] = 0.031
*wR*(*F* ^2^) = 0.099
*S* = 1.17
2191 reflections
168 parameters
All H-atom parameters refined
Δρ_max_ = 0.40 e Å^−3^
Δρ_min_ = −0.24 e Å^−3^

### 

Data collection: *CrysAlis CCD* (Oxford Diffraction (2005 ▶); cell refinement: *CrysAlis RED* (Oxford Diffraction (2005 ▶); data reduction: *CrysAlis RED*; program(s) used to solve structure: *SHELXS97* (Sheldrick, 2008 ▶); program(s) used to refine structure: *SHELXL97* (Sheldrick, 2008 ▶); molecular graphics: *DIAMOND* (Brandenburg, 1999 ▶); software used to prepare material for publication: *SHELXL97*.

## Supplementary Material

Crystal structure: contains datablocks I, global. DOI: 10.1107/S160053680800010X/om2204sup1.cif

Structure factors: contains datablocks I. DOI: 10.1107/S160053680800010X/om2204Isup2.hkl

Additional supplementary materials:  crystallographic information; 3D view; checkCIF report

## Figures and Tables

**Table 1:** Selected torsion angles (°)

O3—B1—C9—C10	6.46 (14)
C5—O4—C10—C9	100.68 (9)
O7—B6—C11—C12	148.79 (9)

**Table 2:** Hydrogen-bond geometry (Å, °)

*D*—H⋯*A*	*D*—H	H⋯*A*	*D*⋯*A*	*D*—H⋯*A*
O2—H2⋯O3^i^	0.865 (17)	1.881 (17)	2.7403 (10)	172.1 (15)
O3—H3⋯O4	0.866 (16)	1.953 (16)	2.6890 (10)	142.1 (14)
O7—H7⋯O8^ii^	0.888 (15)	2.055 (15)	2.8324 (10)	145.6 (13)
O7—H7⋯O4	0.888 (15)	2.317 (14)	2.8573 (10)	119.1 (12)
O8—H8⋯O7^iii^	0.89 (2)	1.88 (2)	2.7615 (10)	172.6 (18)

Symmetry codes: (i) 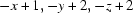; (ii) 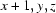; (iii) 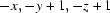.

**Fig. 1.**
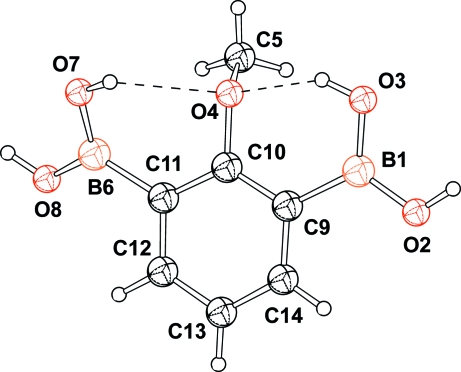
The molecular structure showing the atom-labelling scheme. Intramolecular hydrogen bonds are shown as dashed lines. Displacement ellipsoids for all non-H atoms are drawn at the 50% probability level.

**Fig. 2.**
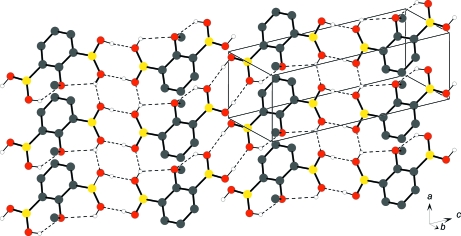
The hydrogen-bonding pattern. Hydrogen bonds are shown as dashed lines.

## supplementary crystallographic information

### Comment

The ability of arylboronic acids to form supramolecular structures *via* hydrogen-bonding interactions of B(OH)_2_ groups is well documented (Rettig & Trotter (1977). The presence of two or more boronic groups in a molecule provides an increased potential for the extended supramolecular organization (Fournier *et al.*, 2003; Maly *et al.*, 2006; Pilkington *et al.*, 1995; Rodríguez-Cuamatzi, Vargas-Díaz & Höpfl (2004). The promising properties of di- and polyboronic acids in crystal engineering prompted us to determine the structure of the title compound.

The molecular structure is shown in Fig. 1. One of two boronic groups is almost coplanar with the benzene ring whereas the second one is significantly twisted (Table 1). The methoxy group is twisted almost perpendicularly with respect to the aromatic ring. Both boronic groups have an *exo*-*endo* conformation. The *endo*-oriented OH groups of both boronic moieties are engaged into intramolecular O—H···O bonds with the methoxy O atom. As a result, a nearly planar six-membered ring is formed by the boronic group coplanar with the benzene ring. This motif has already been observed in structures of related *ortho*-alkoxyarylboronic acids (Yang *et al.*, 2005; Dabrowski *et al.*, 2006; Serwatowski *et al.*, 2006) and seems to be typical. The interaction of the second (twisted) boronic group with the methoxy O atom is much weaker [H7···O4 at 2.317 (14) Å]. The molecules are linked *via* almost linear O—H···O bridges in a "head-to-head, tail-to-tail" fashion, *i.e.*, equivalent groups interact with each other forming two alternate centrosymmetric dimeric motifs. As a result, an infinite, *zig*-*zag* chain is formed (Fig. 2). A similar situation is observed in 1,4-phenylenediboronic acid Rodríguez-Cuamatzi, Vargas-Díaz & Höpfl, 2004) and its tetrahydrate (Rodríguez-Cuamatzi, Vargas-Díaz, Maris, Wuest & Höpfl (2004). However, in the former structure both boronic groups are conformationally equivalent whereas in the latter they are almost coplanar with the aromatic ring. The one-dimensional supramolecular architecture extends through cross-linking O—H···O bonds between twisted boronic groups. As a result a two-dimensional network is formed, aligned parallel to the (01–1) plane. Unlike the structure of related diboronic acids (Maly *et al.*, 2006; Rodríguez-Cuamatzi, Vargas-Díaz & Höpf, 2004), only one boronic group is active as a linker for chains.

In conclusion, the intermolecular hydrogen-bonding interactions of boronic groups are operative to form the chain structure whereas their contribution to further secondary supramolecular organization is strongly affected by competitive intramolecular hydrogen bonds.

### Experimental

A solution of 2,6-dibromoanisole (5.32 g, 20 mmoL, prepared using the published procedure: Dorman, 1966) in Et_2_O (20 ml) was added under argon to a solution of *n*BuLi (10 mol, 4.5 ml, 45 mmol) in THF (60 ml) at 203 K. The mixture was stirred for 30 min at 233 K and then cooled again to 203 K followed by rapid addition of trimethyl borate (5.2 g, 50 mmol). The mixture was stirred for 30 min at 273 K and then it was quenched with HCl (2 *M* solution in ether, 22 ml, 44 mmol). The resultant mixture was concentrated and the residue fractionally distilled *in vacuo* to give 2,6-bis(dimethoxyboryl)anisole as on oil (2.50 g, 50%), b.p. 377–381 K (0.5 Torr). It was hydrolyzed with water (0.9 g, 50 mmol) in acetone (20 ml); the resultant solution was left to evaporate. A remaining crystalline product was filtered and washed with ethyl acetate and hexane to give 1.7 g of the title compound, m.p. > 670 K (with decomposition). ^1^H NMR (acetone-*d*_6 _+ D_2_O): 7.73 (dd, 2 H), 7.08 (t, 1 H), 3.83 (s, 3 H) p.p.m.; ^13^C NMR: 171.0, 138.8, 123.8, 63.2; ^11^B NMR: 29.0 p.p.m..

Crystals suitable for single-crystal X-ray diffraction analysis were grown by slow evaporation of a solution of the acid (0.2 g) in ethyl acetate/acetone/water (20 ml, 10:10:1).

### Refinement

All hydrogen atoms were located in difference syntheses and refined freely.

### Crystal data

**Table:**

C_7_H_10_B_2_O_5_	*Z* = 2
*M**_r_* = 195.77	*F*_000_ = 204
Triclinic, *P*1	*D*_x_ = 1.426 Mg m^−^^3^
*a* = 5.0261 (6) Å	Melting point: 670 K
*b* = 7.6475 (12) Å	Mo *K*α radiation λ = 0.71073 Å
*c* = 12.4535 (19) Å	µ = 0.12 mm^−^^1^
α = 79.010 (13)º	*T* = 100 (2) K
β = 81.898 (12)º	Prismatic, colourless
γ = 77.246 (12)º	0.75 × 0.28 × 0.16 mm
*V* = 455.85 (11) Å^3^	

### Data collection

**Table:**

Kuma KM4 CCD diffractometer	2191 independent reflections
Radiation source: fine-focus sealed tube	1884 reflections with *I* > 2σ(*I*)
Monochromator: graphite	*R*_int_ = 0.012
Detector resolution: 8.6479 pixels mm^-1^	θ_max_ = 28.6º
*T* = 100(2) K	θ_min_ = 2.8º
ω scans	*h* = −6→6
Absorption correction: multi-scan(CrysAlis RED; Oxford Diffraction 2005)	*k* = −10→10
*T*_min_ = 0.91, *T*_max_ = 0.98	*l* = −16→16
8626 measured reflections	

### Refinement

**Table:**

Refinement on *F*^2^	Hydrogen site location: inferred from neighbouring sites
Least-squares matrix: full	All H-atom parameters refined
*R*[*F*^2^ > 2σ(*F*^2^)] = 0.031	*w* = 1/[σ^2^(*F*_o_^2^) + (0.0674*P*)^2^ + 0.0064*P*] where *P* = (*F*_o_^2^ + 2*F*_c_^2^)/3
*wR*(*F*^2^) = 0.099	(Δ/σ)_max_ = 0.001
*S* = 1.17	Δρ_max_ = 0.40 e Å^−^^3^
2191 reflections	Δρ_min_ = −0.24 e Å^−^^3^
168 parameters	Extinction correction: SHELXL, Fc^*^=kFc[1+0.001xFc^2^λ^3^/sin(2θ)]^-1/4^
Primary atom site location: structure-invariant direct methods	Extinction coefficient: 0.074 (12)
Secondary atom site location: difference Fourier map	

### Special details

**Table:**

Geometry. All e.s.d.'s (except the e.s.d. in the dihedral angle between two l.s. planes) are estimated using the full covariance matrix. The cell e.s.d.'s are taken into account individually in the estimation of e.s.d.'s in distances, angles and torsion angles; correlations between e.s.d.'s in cell parameters are only used when they are defined by crystal symmetry. An approximate (isotropic) treatment of cell e.s.d.'s is used for estimating e.s.d.'s involving l.s. planes.
Refinement. Refinement of *F*^2^ against ALL reflections. The weighted *R*-factor *wR* and goodness of fit *S* are based on *F*^2^, conventional *R*-factors *R* are based on *F*, with *F* set to zero for negative *F*^2^. The threshold expression of *F*^2^ > σ(*F*^2^) is used only for calculating *R*-factors(gt) *etc*. and is not relevant to the choice of reflections for refinement. *R*-factors based on *F*^2^ are statistically about twice as large as those based on *F*, and *R*- factors based on ALL data will be even larger.

### Fractional atomic coordinates and isotropic or equivalent isotropic displacement parameters (Å^2^)

**Table:**

	*x*	*y*	*z*	*U*_iso_*/*U*_eq_	
B1	0.2297 (2)	1.01153 (14)	0.88850 (8)	0.0121 (2)	
O2	0.17562 (14)	1.15369 (9)	0.94414 (6)	0.01497 (19)	
O3	0.44802 (13)	0.87075 (9)	0.91170 (6)	0.01494 (18)	
O4	0.27015 (13)	0.71276 (8)	0.76769 (5)	0.01290 (18)	
C5	0.1659 (2)	0.56730 (13)	0.84048 (9)	0.0204 (2)	
B6	−0.0649 (2)	0.70611 (14)	0.59403 (8)	0.0119 (2)	
O7	0.18385 (13)	0.59475 (9)	0.57331 (6)	0.01505 (19)	
O8	−0.28690 (13)	0.67665 (10)	0.55374 (6)	0.01734 (19)	
C9	0.04151 (18)	1.01375 (12)	0.79683 (7)	0.0114 (2)	
C10	0.06518 (18)	0.86766 (12)	0.74123 (7)	0.0108 (2)	
C11	−0.10218 (18)	0.86757 (12)	0.66004 (7)	0.0117 (2)	
C12	−0.30411 (19)	1.02389 (13)	0.63595 (8)	0.0142 (2)	
C13	−0.33351 (19)	1.17323 (13)	0.68851 (8)	0.0154 (2)	
C14	−0.16146 (19)	1.16694 (12)	0.76789 (8)	0.0138 (2)	
H2	0.285 (3)	1.140 (2)	0.9942 (13)	0.042 (4)*	
H3	0.468 (3)	0.793 (2)	0.8674 (14)	0.048 (4)*	
H5A	0.086 (3)	0.6033 (17)	0.9132 (11)	0.029 (3)*	
H5B	0.322 (3)	0.4681 (19)	0.8490 (11)	0.034 (4)*	
H5C	0.019 (3)	0.5290 (19)	0.8100 (12)	0.038 (4)*	
H7	0.322 (3)	0.6297 (19)	0.5953 (12)	0.039 (4)*	
H8	−0.239 (4)	0.591 (3)	0.5115 (15)	0.058 (5)*	
H12	−0.422 (3)	1.0288 (16)	0.5792 (10)	0.023 (3)*	
H13	−0.479 (3)	1.2810 (18)	0.6688 (10)	0.023 (3)*	
H14	−0.189 (2)	1.2703 (16)	0.8053 (10)	0.019 (3)*	

### Atomic displacement parameters (Å^2^)

**Table:**

	*U*^11^	*U*^22^	*U*^33^	*U*^12^	*U*^13^	*U*^23^
B1	0.0127 (5)	0.0138 (5)	0.0112 (5)	−0.0050 (4)	−0.0012 (4)	−0.0024 (4)
O2	0.0170 (4)	0.0154 (4)	0.0149 (4)	−0.0025 (3)	−0.0059 (3)	−0.0060 (3)
O3	0.0150 (4)	0.0160 (4)	0.0163 (4)	−0.0011 (3)	−0.0059 (3)	−0.0080 (3)
O4	0.0131 (3)	0.0111 (3)	0.0149 (3)	−0.0003 (3)	−0.0039 (2)	−0.0039 (3)
C5	0.0258 (5)	0.0139 (5)	0.0208 (5)	−0.0040 (4)	−0.0052 (4)	0.0011 (4)
B6	0.0123 (5)	0.0141 (5)	0.0101 (5)	−0.0033 (4)	−0.0019 (4)	−0.0032 (4)
O7	0.0110 (3)	0.0184 (4)	0.0190 (4)	−0.0020 (3)	−0.0037 (3)	−0.0105 (3)
O8	0.0116 (3)	0.0228 (4)	0.0217 (4)	−0.0018 (3)	−0.0031 (3)	−0.0144 (3)
C9	0.0118 (4)	0.0133 (4)	0.0107 (4)	−0.0045 (3)	−0.0010 (3)	−0.0034 (3)
C10	0.0094 (4)	0.0116 (4)	0.0114 (4)	−0.0021 (3)	−0.0011 (3)	−0.0020 (3)
C11	0.0116 (4)	0.0142 (4)	0.0111 (4)	−0.0038 (3)	−0.0011 (3)	−0.0047 (3)
C12	0.0141 (4)	0.0173 (5)	0.0124 (4)	−0.0030 (4)	−0.0042 (3)	−0.0036 (4)
C13	0.0154 (5)	0.0139 (5)	0.0160 (5)	0.0009 (4)	−0.0046 (3)	−0.0027 (4)
C14	0.0159 (5)	0.0124 (5)	0.0147 (5)	−0.0032 (4)	−0.0023 (3)	−0.0051 (4)

### Geometric parameters (Å, °)

**Table:**

B1—O2	1.3564 (12)	B6—C11	1.5738 (13)
B1—O3	1.3768 (12)	O7—H7	0.888 (15)
B1—C9	1.5774 (13)	O8—H8	0.89 (2)
O2—H2	0.865 (17)	C9—C10	1.3982 (13)
O3—H3	0.866 (16)	C9—C14	1.3993 (13)
O4—C10	1.4075 (11)	C10—C11	1.4036 (12)
O4—C5	1.4397 (12)	C11—C12	1.4009 (13)
C5—H5A	1.001 (13)	C12—C13	1.3920 (13)
C5—H5B	0.965 (14)	C12—H12	0.975 (12)
C5—H5C	0.995 (15)	C13—C14	1.3899 (13)
B6—O8	1.3635 (12)	C13—H13	0.991 (13)
B6—O7	1.3704 (12)	C14—H14	0.967 (12)
			
O2—B1—O3	119.76 (8)	C10—C9—B1	123.17 (8)
O2—B1—C9	118.66 (8)	C14—C9—B1	120.01 (8)
O3—B1—C9	121.57 (8)	C9—C10—C11	123.70 (8)
B1—O2—H2	112.8 (10)	C9—C10—O4	117.99 (8)
B1—O3—H3	110.6 (11)	C11—C10—O4	118.30 (8)
C10—O4—C5	113.05 (7)	C12—C11—C10	116.72 (8)
O4—C5—H5A	111.6 (8)	C12—C11—B6	119.95 (8)
O4—C5—H5B	105.1 (8)	C10—C11—B6	123.29 (8)
H5A—C5—H5B	110.9 (11)	C13—C12—C11	121.55 (8)
O4—C5—H5C	112.4 (9)	C13—C12—H12	119.7 (7)
H5A—C5—H5C	106.8 (11)	C11—C12—H12	118.7 (7)
H5B—C5—H5C	110.0 (12)	C14—C13—C12	119.49 (8)
O8—B6—O7	118.14 (8)	C14—C13—H13	121.7 (7)
O8—B6—C11	119.17 (8)	C12—C13—H13	118.8 (7)
O7—B6—C11	122.67 (8)	C13—C14—C9	121.71 (8)
B6—O7—H7	113.0 (9)	C13—C14—H14	118.3 (7)
B6—O8—H8	111.4 (12)	C9—C14—H14	119.9 (7)
C10—C9—C14	116.81 (8)		
			
O2—B1—C9—C10	−174.50 (8)	C9—C10—C11—B6	177.05 (8)
O3—B1—C9—C10	6.46 (14)	O4—C10—C11—B6	−2.39 (13)
O2—B1—C9—C14	5.35 (14)	O8—B6—C11—C12	−29.98 (13)
O3—B1—C9—C14	−173.69 (8)	O7—B6—C11—C12	148.79 (9)
C14—C9—C10—C11	−0.27 (14)	O8—B6—C11—C10	152.40 (9)
B1—C9—C10—C11	179.58 (8)	O7—B6—C11—C10	−28.83 (14)
C14—C9—C10—O4	179.17 (7)	C10—C11—C12—C13	1.22 (14)
B1—C9—C10—O4	−0.99 (13)	B6—C11—C12—C13	−176.55 (8)
C5—O4—C10—C9	100.68 (9)	C11—C12—C13—C14	−0.88 (15)
C5—O4—C10—C11	−79.86 (10)	C12—C13—C14—C9	−0.10 (15)
C9—C10—C11—C12	−0.64 (14)	C10—C9—C14—C13	0.65 (14)
O4—C10—C11—C12	179.93 (7)	B1—C9—C14—C13	−179.20 (8)

### Hydrogen-bond geometry (Å, °)

**Table:**

*D*—H···*A*	*D*—H	H···*A*	*D*···*A*	*D*—H···*A*
O2—H2···O3^i^	0.865 (17)	1.881 (17)	2.7403 (10)	172.1 (15)
O3—H3···O4	0.866 (16)	1.953 (16)	2.6890 (10)	142.1 (14)
O7—H7···O8^ii^	0.888 (15)	2.055 (15)	2.8324 (10)	145.6 (13)
O7—H7···O4	0.888 (15)	2.317 (14)	2.8573 (10)	119.1 (12)
O8—H8···O7^iii^	0.89 (2)	1.88 (2)	2.7615 (10)	172.6 (18)

Symmetry codes: (i) −*x*+1, −*y*+2, −*z*+2; (ii) *x*+1, *y*, *z*; (iii) −*x*, −*y*+1, −*z*+1.
